# Endothelial Cells Derived from the Blood-Brain Barrier and Islets of Langerhans Differ in their Response to the Effects of Bilirubin on Oxidative Stress Under Hyperglycemic Conditions

**DOI:** 10.3389/fphar.2012.00131

**Published:** 2012-07-13

**Authors:** Jaime Kapitulnik, Clara Benaim, Shlomo Sasson

**Affiliations:** ^1^Department of Pharmacology, Institute of Drug Research, School of Pharmacy, The Hebrew University of JerusalemJerusalem, Israel

**Keywords:** bilirubin, glucose, blood-brain barrier, jaundice, diabetes, apoptosis, oxidative stress, reactive oxygen species

## Abstract

Unconjugated bilirubin (UCB) is a neurotoxic degradation product of heme. Its toxic effects include induction of apoptosis, and ultimately neuronal cell death. However, at low concentrations, UCB is a potent antioxidant that may protect cells and tissues against oxidative stress by neutralizing toxic metabolites such as reactive oxygen species (ROS). High glucose levels (hyperglycemia) generate reactive metabolites. Endothelial cell dysfunction, an early vascular complication in diabetes, has been associated with hyperglycemia-induced oxidative stress. Both glucose and UCB are substrates for transport proteins in microvascular endothelial cells of the blood-brain barrier (BBB). In the current study we show that UCB (1–40 μM) induces apoptosis and reduces survival of bEnd3 cells, a mouse brain endothelial cell line which serves as an *in vitro* model of the BBB. These deleterious effects of UCB were enhanced in the presence of high glucose (25 mM) levels. Interestingly, the bEnd3 cells exhibited an increased sensitivity to the apoptotic effects of UCB when compared to the MS1 microcapillary endothelial cell line. MS1 cells originate from murine pancreatic islets of Langerhans, and are devoid of the barrier characteristics of BBB-derived endothelial cells. ROS production was increased in both bEnd3 and MS1 cells exposed to high glucose, as compared with cells exposed to normal (5.5 mM) glucose levels. While UCB (0.1–40 μM) did not alter ROS production in cells exposed to normal glucose, relatively low (“physiological”) UCB concentrations (0.1–5 μM) attenuated ROS generation in both cell lines exposed to high glucose levels. Most strikingly, higher UCB concentrations (20–40 μM) increased ROS generation in bEnd3 cells exposed to high glucose, but not in similarly treated MS1 cells. These results may be of critical importance for understanding the vulnerability of the BBB endothelium upon exposure to increasing UCB levels under hyperglycemic conditions.

## Introduction

**Figure 1:**
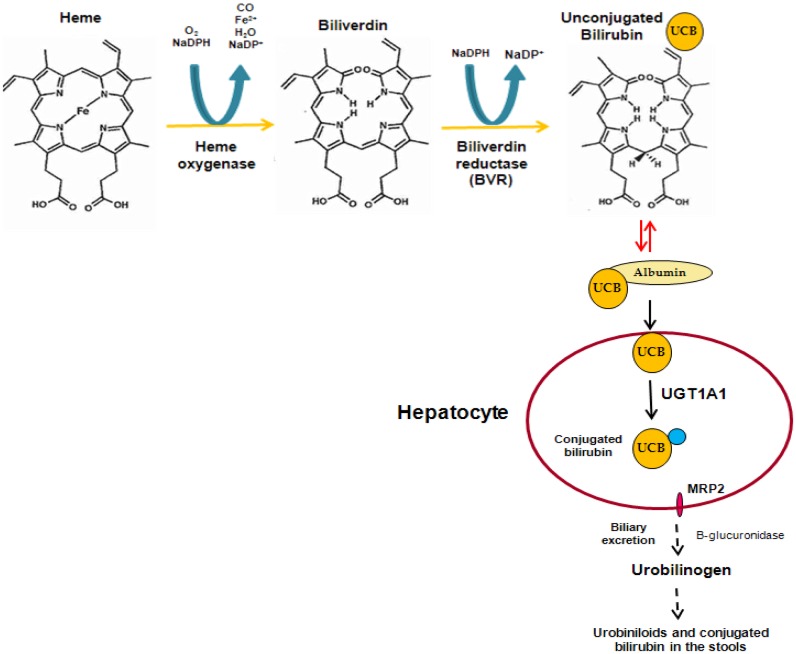
**Formation and elimination of unconjugated bilirubin**.

Bilirubin is a linear tetrapyrrole that is formed during the process of heme degradation. Heme is released from a series of hemeproteins, including hemoglobin and cytochromes P450, and metabolized by heme oxygenase to form carbon monoxide, biliverdin, and free iron. Biliverdin is subsequently transformed to unconjugated bilirubin (UCB) by biliverdin reductase. UCB binds to plasma albumin, which transports it to the liver, where it is conjugated to hydrophilic acceptors. The major conjugates are bilirubin glucuronides formed by UDP-glucuronosyltransferase 1A1 (UGT1A1). These polar derivatives are thereafter excreted in the bile (Figure 1).

Unconjugated bilirubin is a molecule which behaves as a “double-edged sword” (Kapitulnik, 2004); it exerts both cytotoxic and cytoprotective effects which are dose- and target-dependent. When the blood levels of UCB are excessively elevated and surpass the capacity of albumin for high-affinity binding of UCB, the unbound (free) fraction of the pigment increases. Free UCB can easily enter cells by passive diffusion and cause toxicity. The most vulnerable target is the central nervous system (CNS). UCB binds to discrete brain areas, such as the basal ganglia (kernicterus), and produces a wide array of neurological deficits collectively known as bilirubin encephalopathy.

The cytotoxic effects of UCB include programmed cell death (apoptosis) of a variety of cell types such as neurons (Grojean et al., 2000; Rodrigues et al., 2002), aortic smooth muscle cells (Liu et al., 2002), brain microvascular endothelial cells (Akin et al., 2002), and hepatoma cells (Seubert et al., 2002).

The pioneering studies conducted in the laboratory of Bruce Ames (Stocker et al., 1987a,b) introduced the concept that UCB, which was until then regarded as a toxic waste product of heme catabolism, possesses a beneficial role at low (“physiological”) plasma concentrations (<1 mg/dl, <17 μM) by acting as a potent antioxidant that scavenges reactive oxygen species (ROS). Almost two decades ago it was suggested that moderately elevated plasma UCB levels may reduce the risk of coronary artery disease (Schwertner et al., 1994). Indeed, individuals with Gilbert syndrome, a mild form of hyperbilirubinemia, who display moderately increased plasma UCB concentrations (~40 μM) due to a decreased UGT1A1 activity, show a lower risk of cardiovascular and related diseases such as diabetes, as compared with normobilirubinemic subjects (reviewed by Lin et al., 2010; Vitek, 2012).

High glucose (hyperglycemia) augments production of ROS, which play a major role in the etiology of diabetes complications, such as endothelial dysfunction (Guzik et al., 2002) and astrocyte activation in the CNS (Wang et al., 2012). Of interest are the findings that ROS generated by hyperglycemia cause apoptosis of vascular endothelial cells (Hulsmans et al., 2012) and are responsible for the endothelial cell sloughing associated with hyperglycemia (Rodella et al., 2006).

Glucose is an essential nutrient for brain cells and crosses the blood-brain barrier (BBB) via the influx glucose transporter-1 (GLUT1). The entry of the neurotoxic UCB into the brain is prevented by the efflux transporter P-glycoprotein (P-gp). Thus, it was of great interest to examine the interplay between UCB and glucose in BBB-derived endothelial cells. In the current study we examined the effects of low (“physiological”) and moderately elevated UCB concentrations (0.1–40 μM), alone or in combination with high glucose, on apoptosis and cellular ROS levels in bEnd3 cells, which are a model for studying BBB characteristics. The responses of these cells to UCB and glucose were compared with those of MS1 cells, microvascular endothelial cells derived from pancreatic islets of Langerhans. The latter cells do not exhibit barrier characteristics and may serve as a model for studying free glucose passage into pancreatic islets.

## Materials and Methods

### Chemicals

Dulbecco’s modified Eagle’s medium (DMEM), fetal bovine serum (FBS), HEPES, SDS 10% (wt/vol) solution, l-glutamine solution (2 mM), penicillin (100 U/ml) and streptomycin (0.1 mg/ml) solution, sodium pyruvate, and trypsin were purchased from Biological Industries (Beth-Haemek, Israel). Micro BCA™ protein assay kit, bovine serum albumin (BSA; fraction V), DMSO, PBS, NaOH, and staurosporine solution were purchased from Sigma-Aldrich (Rehovot, Israel). UCB was purchased from MP Biomedicals (Santa Ana, CA, USA), hydrogen peroxide 30% (wt/vol) from J. T. Baker (Phillipsburg, NJ, USA), 5- (and 6-) chloromethyl-2′,7′-dichlorodihydrofluorescein diacetate (CM-H_2_DCFDA) from Molecular Probes (Eugene, OR, USA), and D(+) glucose anhydrous from Riedel-de Haën (Seelze, Germany).

### Cell culture

The bEnd3 microvascular endothelial cell line is derived from mouse brain. These cells display structural and functional barrier properties owing to their high expression of tight junction proteins and high transendothelial resistance, thus resembling the BBB (Brown et al., 2007; Li et al., 2010). The MS1 microvascular endothelial cell line is derived from mouse pancreatic islets. These cells are highly fenestrated and do not show barrier characteristics (Konstantinova and Lammert, 2004). The bEnd3 cells were a gift from Dr. P. Lazarovici (School of Pharmacy, Hebrew University of Jerusalem), and MS1 cells were purchased from American Type Culture Collection (ATCC, Manassas, VA, USA).

Both cell types were grown in plastic flasks in DMEM culture medium supplemented with 1% (vol/vol) antibiotics, 1% (vol/vol) l-glutamine, 10% (vol/vol) FBS, and 5.5 mM glucose (37°C, 5% CO_2_). After reaching confluency, cells were plated to 12 well-plates in 2 ml of fresh culture medium per well. The culture medium was replaced 24 h after seeding with fresh medium containing 5.5 or 25 mM glucose. After an additional 24 h-incubation period, the culture medium was replaced with fresh medium containing increasing concentrations of UCB. A fresh stock solution of UCB (40 mM, in 0.1 M NaOH) was prepared for each experiment. Serial dilutions of the UCB stock solution were made in fresh culture medium containing 20 mM HEPES, to keep a stable pH of 7.5–7.6, and 5.5 or 25 mM glucose. Cells were further incubated for an additional 24 h-period. Addition of UCB and further handling of cell cultures was done in dim light.

### Cell number determination

Cells were detached from the plate with 0.25 ml of a solution of trypsin (1:2000). Volume was adjusted to 0.5 ml with culture medium and cells were counted in a hemacytometer.

### Apoptosis

Apoptosis was assayed using the FAM-FLICA™ Poly Caspases Assay Kit (ImmunoChemistry Technologies, LLC, Bloomington, MN, USA). This assay detects the presence of caspase activity in apoptotic cells by quantifying the intensity of fluorescence in cells exposed to a membrane-permeant, Fluorescent Labeled Inhibitor of Caspases (FLICA) probe. The fluorescent tag is carboxyfluorescein. This probe binds covalently with the active caspases and is retained only in apoptotic cells.

After incubation with increasing concentrations of UCB, the cells were analyzed according to the manufacturer’s instructions. Briefly, cells were trypsinized, followed by centrifugation and resuspension in culture medium. Triplicate samples of 2.5 × 10^6^ cells from each treatment were transferred into plastic tubes and incubated in the dark with the carboxyfluorescein-FLICA solution for 1 h (37°C, 5% CO_2_). Cell suspensions were thereafter washed twice with wash buffer and resuspended in PBS, keeping a final uniform cell density (2.5 × 10^6^ cells/sample). One hundred microliters of each cell suspension was placed into each of three wells of a black microtiter plate for measurement of fluorescence intensity (excitation: 490 nm; emission: 520 nm). Thus, triplicate readings were obtained for each triplicate caspase sample. A positive control was prepared by incubating naive (untreated) cells with 1 μM staurosporine for 3 h. Staurosporine induces apoptosis by caspase activation (Thuret et al., 2003).

Caspase activity was expressed as relative fluorescence units (RFU), calculated by dividing the individual fluorescence values for the different treatments by that obtained for cells exposed to 5.5 mM glucose in the absence of UCB (the latter were assigned a value of 1). Experiments were repeated at least three times.

### Reactive oxygen species

Reactive oxygen species production was examined using the cell-permeant probe 5- (and 6-) chloromethyl-2′,7′-dichlorodihydrofluorescein diacetate (CM-H_2_DCFDA). This dye enters the cell by passive diffusion, undergoes hydrolysis to generate chloromethyl-dichlorodihydrofluorescein (CM-H_2_DCF), which remains trapped intracellularly. Upon oxidation of CM-H_2_DCF by ROS, the fluorescent product chloromethyl-dichlorofluorescein (CM-DCF) is formed (Kehrer and Paraidathathu, 1992). After incubation with UCB, cells were rinsed three times with PBS and incubated with CM-H_2_DCFDA for 30 min (37°C, 5% CO_2_). The cells were thereafter washed three times with PBS and solubilized with 600 μl SDS 0.1% (wt/vol). One hundred microliters of each sample was placed into each of three wells of black microtiter plates, and fluorescence intensity was measured (excitation: 490 nm; emission: 520 nm). A positive control was prepared by incubating naive (untreated) cells with 1 mM H_2_O_2_ for 2 h.

Reactive oxygen species production was normalized to protein content. Twenty microliters aliquots of each sample were placed into each of three wells of 96-multiwell plates. Volume was adjusted to 150 μl with SDS 0.1%, and 150 μl of Micro BCA working reagent was added. The plates were covered and incubated for 2 h (37°C, 5% CO_2_), and the colored product was quantitated by measuring absorbance at 562 nm.

Reactive oxygen species production is expressed as RFU, calculated by dividing the individual fluorescence values for the different treatments (normalized to protein content) by that obtained for cells exposed to 5.5 mM glucose in the absence of UCB (the latter were assigned a value of 1). Experiments were repeated at least three times.

### Statistical analysis

The experiments were carried out in replicates of *n* ≥ 3 and results are presented as Mean ± Standard Deviation. Statistical differences were evaluated using the Student’s two-tailed *t* test. A “*p*” value of ≤0.05 indicates statistical significance.

## Results

### Apoptotic effects of bilirubin and/or glucose

**Figure 2:**
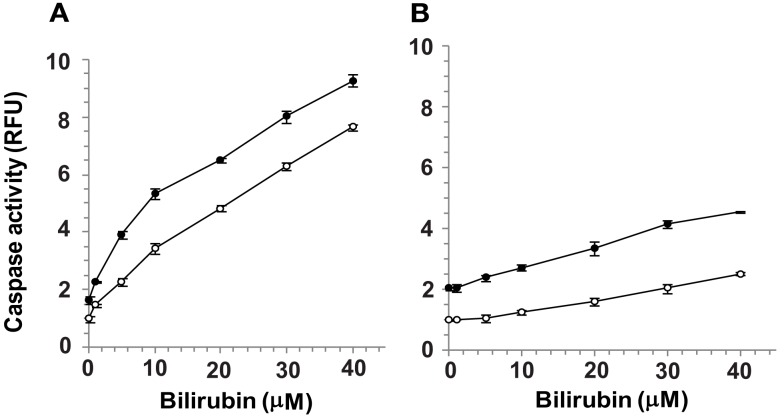
**Apoptotic effects of UCB and glucose in microvascular endothelial cells**. Confluent cultures of bEnd3 **(A)** or MS1 **(B)** cells were incubated for 48 h with culture medium containing normal (5.5 mM; -○-) or high (25 mM; -•-) glucose, in the absence or presence of increasing concentrations of UCB added during the last 24 h of incubation. Caspase activity was determined using the carboxyfluorescein-FLICA probe (see Materials and Methods) and was expressed as relative fluorescence units (RFU), calculated by dividing the individual fluorescence values for the different treatments by that obtained for cells exposed to 5.5 mM glucose in the absence of UCB (the latter were assigned a value of 1). Experiments were repeated at least three times. The results of a representative experiment are shown as Mean ± Standard Deviation. Values for the bEnd cells at all UCB concentrations differ from the values obtained in the absence of UCB (statistically significant at *p* < 0.05). Values for the MS1 cells at UCB concentrations of 10–40 μM differ from the values obtained in the absence of UCB (statistically significant at *p* < 0.05).

Confluent cultures of bEnd3 and MS1 cells were incubated for 48 h with culture medium containing normal (5.5 mM) or high (25 mM) glucose, in the absence or presence of increasing concentrations of UCB added during the last 24 h of incubation. UCB (1–40 μM) caused a concentration-dependent increase of caspase activity in both cell types (Figure 2). This effect was more pronounced in bEnd3 than in MS1 cells. The 40 μM concentration of UCB caused a 7.6-fold increase in caspase activity in the normal glucose-containing cultures (Figure 2A), as compared with a 2.5-fold increase in MS1 cells under similar conditions (Figure 2B). Noteworthy, in contrast with the marked apoptotic effect of UCB in bEnd3, but not in MS1 cells, exposure of both cell types to 1 μM staurosporine caused a 6.2-fold increase in caspase activity. Elevation of the glucose concentration to 25 mM in the absence of UCB, increased caspase activity nearly twofold in both cell types, and further increased the effect of 40 μM UCB to 9.3- and 4.5-fold for bEnd3 and MS1 cells, respectively (Figures 2A,B). It should be noted that the relative degree of apoptosis in high glucose-containing bEnd3 cultures was more pronounced for the lower (1–10 μM) than for the higher (20–40 μM) UCB concentrations (Figure 2A). UCB also reduced the number of viable bEnd3 cells, and high glucose increased this effect (Table 1). As described above for the caspase activity determinations, the MS1 cells were also less sensitive to the cytotoxic effects of UCB. The fraction of non-viable/apoptotic cells was less than 1% for all treatments of both cell lines (data not shown).

**Table 1:** **Viability of microvascular endothelial cells exposed to UCB in normal or high glucose-containing culture media**.

UCB (μM)	bEnd3 cells		MS1 cells	
	5.5 mM glucose	25 mM glucose	5.5 mM glucose	25 mM glucose
0	100	100	100	100
10	94.5 ± 1.0	91.6 ± 1.7	94.8 ± 1.6	95.1 ± 3.5
40	85.0 ± 0.7	77.5 ± 2.1	90.3 ± 1.0	84.5 ± 0.5

*Cells were counted at the end of incubations with UCB in normal (5.5 mM) or high (25 mM) glucose-containing media. The fraction of non-viable/apoptotic cells was less than 1% for all treatments of both cell lines (data not shown). Results are expressed as percentages of viable cells, and are presented as Mean ± Standard Deviation of triplicate samples. Numbers of cells in the absence of UCB are taken as 100% values: bEnd3 cells in normal glucose – 1.47 × 10^6^ cells/ml; in high glucose – 1.78 × 10^6^ cells/ml. MS1 cells in normal glucose – 1.55 × 10^6^ cells/ml; in high glucose – 1.23 × 10^6^ cells/ml*.

### Effects of UCB on the generation of ROS in endothelial cells cultured in normal and high glucose-containing media

Given the differential sensitivity of bEnd3 vs. MS1 cells toward the apoptotic effect of UCB, and the involvement of ROS in apoptosis of endothelial cells, it was of a great interest to determine whether these two cell types also differ in their capacity to produce ROS in response to either UCB or high glucose, or to their combination.

**Figure 3:**
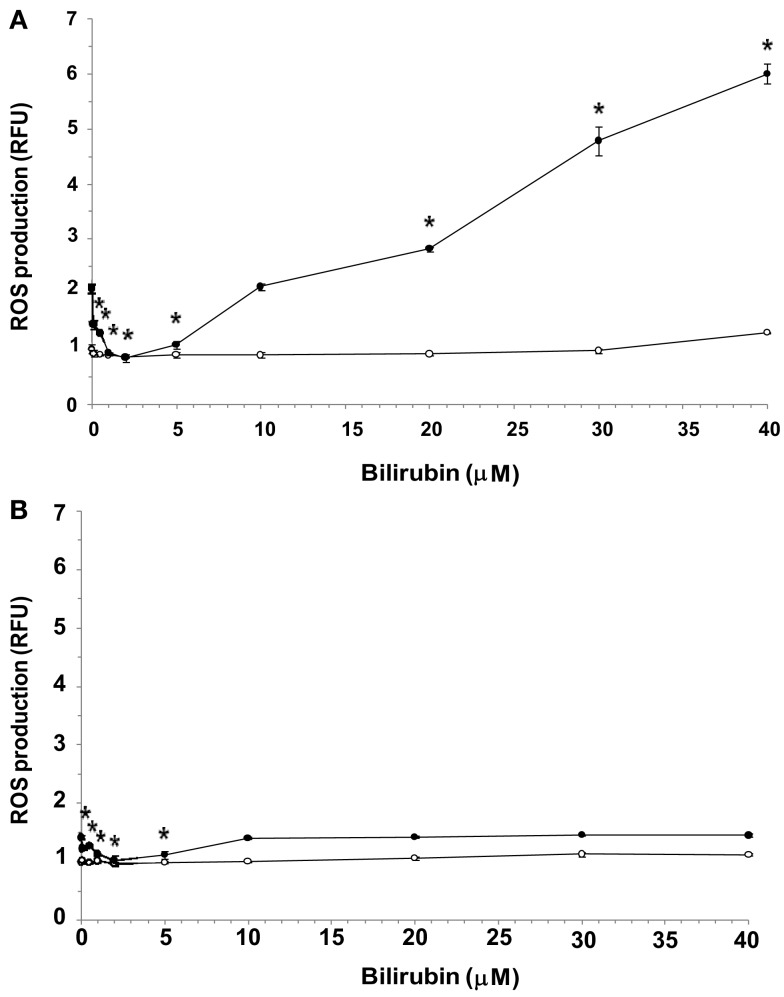
**Effects of UCB on the generation of ROS in microvascular endothelial cells cultured in normal or high glucose-containing media**. Confluent cultures of bEnd3 **(A)** or MS1 **(B)** cells were incubated for 48 h with culture medium containing normal (5.5 mM; -○-) or high (2 mM; -•-) glucose, in the absence or presence of increasing concentrations of UCB added during the last 24 h of incubation. ROS production was assessed using the cell-permeant probe CM-H_2_DCFDA, as described in Materials and Methods. ROS production was normalized by determining protein content and is expressed as relative fluorescence units (RFU), calculated by dividing the individual fluorescence values for the different treatments by that obtained for cells exposed to 5.5 mM glucose in the absence of UCB (the latter were assigned a value of 1). Experiments were repeated at least three times. The results of a representative experiment are shown as Mean ± Standard Deviation. *Statistically significant difference from 0 μM UCB at *p* < 0.05.

Exposure of both bEnd3 and MS1 cells to increasing concentrations of UCB in normal glucose-containing medium had no significant effect on ROS production (Figures 3A,B, respectively). In the bEnd3 cells, elevation of the glucose concentration to 25 mM approximately doubled ROS production in the absence of UCB. The combined exposure of bEnd3 cells to UCB (0.1–40 μM) and high glucose had a biphasic effect on ROS production (Figure 3A). UCB (0.1–5 μM) caused an initial decrease in ROS production: similar ROS values were obtained at 25 and 5.5 mM glucose in the presence of 2 μM UCB. Higher UCB concentrations (20–40 μM) markedly elevated ROS production in the presence of 25 mM glucose.

Exposure of MS1 cells to high glucose caused a minor elevation in ROS production (about 40%) in the absence of UCB (Figure 3B). Again, low (0.1–5 μM) concentrations of UCB abolished this increase, as shown for the bEnd3 cells. However, in contrast to the latter cells, increasing further the UCB concentration (up to 40 μM) did not increase ROS production in MS1 cells above the value obtained in the absence of UCB. In a control experiment, 1 mM H_2_O_2_ caused 1.5- and 1.6-fold increases in ROS production in bEnd3 and MS1 cells, respectively.

## Discussion

The CNS is extremely sensitive to UCB, particularly in the immediate postnatal period. UCB-induced neurotoxicity is affected by the interplay between neurons and glial cells (Silva et al., 2011). Relatively low UCB levels may cause apoptotic cell death, particularly in immature neurons (Grojean et al., 2000; Rodrigues et al., 2002).

We show here that endothelial cells derived from the brain microvasculature exhibit a marked and dose-dependent apoptotic response to UCB, as compared with a weaker response of microvascular endothelial cells obtained from pancreatic islets of Langerhans. Apoptosis in the former cells occurs already at UCB concentrations which are considered “physiological” in humans, namely 1–10 μM, and is accompanied by a reduction in the number of viable cells remaining in culture after a 24 h-exposure to UCB. To our knowledge, this is the first report on a greater sensitivity to UCB of brain-derived microvascular endothelial cells, when compared with their counterparts in other microvessels.

The brain-derived bEnd3 cells are useful for studying BBB function *in vitro* since they display structural and functional barrier properties owing to their high expression of tight junction proteins and high transendothelial resistance (Brown et al., 2007; Li et al., 2010). Glucose enters these cells via the GLUT1 transporter. In contrast, the MS1 cells are highly fenestrated and lack barrier characteristics (Konstantinova and Lammert, 2004). Glucose passage through pancreatic islet microvascular endothelium is free and elicits the release of insulin from β-cells in a glucose concentration-dependent manner.

Increasing the glucose concentration of the culture medium from 5.5 to 25 mM in cultures of both endothelial cell types approximately doubled the caspase activity in the absence of UCB. The combination of a high glucose level and increasing UCB concentrations further increased the apoptotic response of the cells, suggesting that UCB and hyperglycemia induce apoptosis in these cells by independent mechanisms. In developing rat brain neurons, UCB facilitates glutamate-mediated apoptosis through the activation of *N*-methyl-d-aspartate receptors (Grojean et al., 2001) or by directly disrupting the integrity of the mitochondrial membrane, thus inducing mitochondrial depolarization and Bax translocation (Rodrigues et al., 2002). On the other hand, high glucose-mediated apoptosis of vascular endothelial cells results from increased ROS production (van den Oever et al., 2010; Hulsmans et al., 2012).

Under normal glucose conditions (5.5 mM glucose) there was no significant effect of UCB (0.1–40 μM) on ROS production in either cell type, suggesting that ROS are not responsible for UCB-mediated apoptosis in microvascular endothelial cells. In contrast, in the absence of UCB, high glucose levels induced a 2.1-fold increase in ROS production in the brain-derived cells and a 1.4-fold increase in the islet-derived cells.

Most strikingly, the two cell types studied showed different patterns of ROS production in response to the combined exposure to UCB and high glucose (Figures 3A,B). Such an exposure of brain-derived bEnd3 cells affected ROS production in a biphasic manner. Low (“physiological”) concentrations of UCB (0.1–2 μM) attenuated high glucose-induced ROS production, while higher UCB concentrations markedly elevated ROS production under these conditions (Figure 3A). A similar initial decrease in ROS production was observed in the MS1 islet-derived cells exposed to UCB and high glucose. In both cell types the levels of ROS produced at 10 μM UCB resembled the ROS levels measured in the absence of UCB. However, contrary to the bEnd3 cells, further increases in UCB concentration did not cause further changes in ROS production in the MS1 cells (Figure 3B).

Thus, in the presence of high glucose, which by itself is an ROS generator, low UCB concentrations exhibit a protective effect by counteracting the excessive production of ROS in microvascular endothelial cells. The bEnd3 cells responded to higher UCB concentrations by exacerbating the high glucose-mediated oxidative stress, whereas the MS1 cells tolerated well this condition and did not increase further ROS production. These disparate responses suggest that hyperglycemia might compromise the function of the BBB during hyperbilirubinemia, while islets of Langerhans are more resistant to hyperbilirubinemia, and thus may preserve their insulin secreting capacity in response to hyperglycemia. Interestingly, hyperbilirubinemic Gunn rats do not develop diabetes in response to streptozotocin exposure, as compared to normobilirubinemic animals (Fu et al., 2010). Moreover, insulin secretion by the pancreatic islets was preserved in these hyperbilirubinemic animals.

Our data suggest that UCB *per se* does not affect ROS production in microvascular endothelial cells under normal glucose conditions. However, although ROS production under high glucose conditions was diminished by low UCB concentrations in both microvascular cell types, UCB-mediated apoptosis of these cells was not affected by this decrease in ROS production. These findings support a general concept that as much as ROS play a key role in inducing apoptosis, there are other pro-apoptotic factors that act independently of the redox state of cells.

## Conflict of Interest Statement

The authors declare that the research was conducted in the absence of any commercial or financial relationships that could be construed as a potential conflict of interest.
